# Absence of Correlation between IL-28B Gene Polymorphisms and the Clinical Presentation of Chronic Hepatitis B in an Amazon Brazilian Population

**DOI:** 10.1155/2014/534534

**Published:** 2014-04-10

**Authors:** Simone Regina Souza da Silva Conde, Luciana L. Rocha, Vanessa M. Ferreira, Julius Caesar Mendes Soares Monteiro, Nathália Karla Fonseca Filgueiras, Pedro Alves de Almeida Lins, Bruna Tereza Silva dos Santos, Felipe Bonfim Freitas, Ednelza da Silva Graça, Sâmia Demachki, Marialva Tereza Ferreira de Araújo, Ricardo Ishak, Antonio C. R. Vallinoto

**Affiliations:** ^1^School of Medicine, Institute of Health Sciences, Federal University of Pará, Umarizal, 66050-380 Belém, PA, Brazil; ^2^Holy House of Mercy Foundation of Pará, Umarizal, 66050-380 Belém, PA, Brazil; ^3^Virology Laboratory, Institute of Biological Sciences, Federal University of Pará, Guamá, 66075-110 Belém, PA, Brazil

## Abstract

*Objective*. The present study investigated the prevalence of the IL-28B polymorphisms rs12979860 and rs8099917 in chronic hepatitis B patients from a case study in Eastern Amazonia.* Methods*. In total, 65 chronically infected HBV patients and 97 healthy subjects who were anti-HBc and anti-HBs positive (control group) were evaluated between May 2011 and December 2012. The groups of patients were designated as inactive carriers, chronic hepatitis without cirrhosis, and chronic hepatitis with cirrhosis based on clinical, pathological, biochemical, hematological, and virological variables. The patients were genotyped using quantitative real-time PCR.* Results*. The frequencies of the rs12979860 polymorphism were similar between the infected group (32.3% CC, 41.5% CT, and 26.2 TT) and the control population (35% CC, 47.4% CT, and 17.6% TT), and the frequencies of the rs8099917 polymorphism (7.7% GG, 35.4% GT, and 56.9% TT versus 7.2% GG, 35.1% GT, and 57.7% TT) were also similar in both groups. The associations between the rs12979860 and rs8099917 polymorphisms and the clinical manifestations were not statistically significant.* Conclusion*. In conclusion, these polymorphisms had a similar distribution between infected and control groups, indicating that they were not associated with susceptibility and the clinical evolution of hepatitis B in the examined population.

## 1. Introduction 

Hepatitis B virus (HBV) infections are a serious global public health problem. An estimated two billion people have already come into contact with this virus, and approximately 350 million individuals are chronic carriers worldwide. Over one million deaths from this virus are reported annually [1–3].

The endemicity of HBV in Brazil is quite heterogeneous; the disease is the most prevalent in the north region of the country that encompasses Western Amazonia, especially in the zone that covers the states of Acre, Amazonas, Rondônia, and Roraima [4, 5]. In 2011, there were 172 confirmed cases of hepatitis B in the state of Pará, representing approximately 9% of the cases in the north region [6].

HBV infection results in a wide spectrum of clinical manifestations, ranging from an acute self-limiting disease to chronic disease. It is possible to detect inactive carriers and chronic hepatitis patients with or without cirrhosis, which are diseases that result in approximately 500,000 deaths annually worldwide due to liver failure and hepatocellular carcinoma [7–9].

One important factor related to the different clinical manifestations of the disease is interleukin-28B (IL-28B), a cytokine discovered in 2003 that is part of the *γ* interferon family [10]. The gene for IL-28B is located on the long arm of chromosome 19 at position 19q13.13 [11, 12]. All the subtypes of interferon-*γ* play an important role in antiviral immunity, especially in the IL-28B-mediated antiviral defense against hepatotropic viruses, such as HBV and hepatitis C virus (HCV) [13, 14].

Single nucleotide polymorphisms (SNPs) have been found in the IL-28B gene at positions rs12979860 and rs8099917, which are located 3 kilobases upstream of the gene and have recently been associated with the response of certain individuals to viral infections [15, 16].

The IL-28B polymorphisms have been associated with both the spontaneous clearance of HCV and the response to antiviral treatment, which play a key role in the resolution of the infection. Considering that HBV and HCV have similar natural histories, levels of pathogenesis, and modes of transmission, genetic variants of IL-28B may also have a functional role during chronic HBV infection [12].

This study was prompted by the lack of information on the relationship between the genetic profile of IL-28B and HBV infections in Eastern Amazonia, an endemic area for this virus. Therefore, the primary aim of this study was to examine a possible association between the polymorphisms rs12979860 and rs8099917, HBV, and the clinical manifestations of this infection.

## 2. Materials and Methods 

### 2.1. Study Population

This cross-sectional, observational, analytical, and descriptive study was performed in an outpatient clinic for liver diseases at the Holy House of Mercy Foundation of Pará and the Virology Laboratory at the Institute of Biological Sciences, Federal University of Pará in the city of Belém, PA, Brazil.

The study population included 65 patients with chronic HBV whose samples were collected from October 2011 to June 2012. The study included patients who were HBsAg positive for more than six months, with or without a positive HBeAg test, older than 18 years of age, and either male or female. Patients were excluded who had coinfections (human immunodeficiency virus (HIV) or HCV), had previously received treatment, and did not agree to participate.

The control population included 97 healthy subjects of both sexes, older than 18 years of age, who had previously exhibited spontaneous HBV seroconversion (anti-HBc and anti-HBs positive) but were negative for HCV and HIV infections.

### 2.2. Data Collection

Demographic variables including gender, age, birthplace, occupation, educational level, and marital status were examined. The patients were clinically classified into two groups according to clinical, sonographic, endoscopic, and histopathological criteria when possible as follows:

- inactive carriers of HBV (*n* = 26): characterized by consistently normal alanine aminotransferase (ALT) and aspartate aminotransferase (AST) levels over a minimum period of one year, HBeAg negative, anti-HBeAg positive, HBV-DNA < 2000 IU/mL, or/and significant histopathological lesions (*A* ≤ 1 and *F* ≤ 1, METAVIR classification);
- chronic hepatitis B carriers with (*n* = 12) or without cirrhosis (*n* = 27): characterized by clinical changes, liver tests, HBeAg positive or negative, HBV-DNA > 2000 IU/mL, and significant histopathological lesions (*F* ≥ 2 and <4, METAVIR classification); the diagnosis of cirrhosis included signs of hepatocellular failure, portal hypertension, or liver histopathology with a fibrosis score of 4 based on the METAVIR classification.

### 2.3. Laboratory Variables

Routine laboratory tests (blood count, AST, ALT, and gamma-glutamyl transpeptidase) and enzyme immunoassays to test for markers of infection (HBsAg, HBeAg, anti-HBc, anti-HBs, and anti-HCV) were performed at the Laboratory of Clinical Analysis at the Holy House of Mercy Foundation of Pará. Anti-HIV tests and the quantification of the HBV viral load (HBV-PCR DNA quantification, Amplicor, ROCHE) were performed at the Central Laboratory of the Health Department of the State of Pará.

The data considered for this study included the tests performed closest to the collection period for the untreated patients and the tests performed just prior to antiviral therapy for the patients.

### 2.4. Collection and Sample Processing

Blood samples were taken for plasma and cell collection using vacuum collection tubes containing 5 mL of EDTA as an anticoagulant. The samples were subsequently sent to the Virology Laboratory at the Institute of Biological Sciences/Federal University of Pará, where aliquots of plasma and leukocytes were stored at −70°C until use.

### 2.5. DNA Extraction

Total DNA was extracted from peripheral blood leukocytes at the Virology Laboratory at the Institute of Biological Sciences, Federal University of Pará, using a Puregene kit (Gentra Systems, Inc., USA) following the protocol for cell lysis, protein precipitation, DNA precipitation, and rehydration. The DNA samples were stored at −20°C until use.

### 2.6. Genotyping the IL-28B Polymorphisms

Real-time PCR (qPCR) was used to genotype the rs12979860 and rs8099917 SNPs using TaqMan Gene Expression Assay AHCS19G and TaqMan Gene Expression Assay C__11710096_10 kits (Applied Biosystems, Foster City, CA, USA), respectively, following the technical procedures recommended by the manufacturer.

For the rs12979860 polymorphism, a custom-designed assay provided by Applied Biosystems was used. The following primer sequences were used in these assays: forward primer, 5′-GCCTGTCGTGTACTGAACCA-3′; reverse primer, 5′-GCGCGGAGTGCAATTCAAC-3′; allele C (VIC), 5′-TGGTTCGCGCCTTC-3′; allele T (FAM), 5′-CTGGTTCACGCCTTC-3′. For the rs8099917 polymorphism, an assay predesigned by Applied Biosystems was used. The sequence context was 5′-TTTTGTTTTCCTTTCTGTGAGCAAT[G/T]TCA CCCAAATTGGAACCATGCTGTA-3′, in which allele G = VIC and allele T = FAM.

The PCR reactions were prepared using TaqMan Universal PCR Master Mix components (Applied Biosystems), which contained nucleotides, buffer, UNG, AmpliTaq, and a passive reference dye (ROX). The reaction mixture contained 5.0 *μ*L of Master Mix, 3.5 *μ*L of H_2_O, 0.5 *μ*L of IL-28B assay components (primer set and probe), and 1.0 *μ*L of DNA from each sample. The final volume for each reaction was 10 *μ*L.

A Step One Plus Real-Time PCR System (Applied Biosystems) was used to perform the qPCR experiments using the following cycling protocol: one cycle of 60°C for 2 minutes, one cycle of 95°C for 10 minutes, and 50 cycles of 95°C for 15 seconds and 60°C for 20 seconds.

The program StepOne v2.2 (Applied Biosystems) was used to interpret the reaction results, using the graphical representation of the VIC and FAM fluorophore emissions with respect to constitutive ROX emissions.

### 2.7. Histopathological Variables

Upon providing informed consent, the patients who had prothrombin activity less than or equal to 70% and platelets counts above 100,000 cells/mm^3^ underwent a liver biopsy.

The liver biopsies were performed using a Trucut needle and were guided by ultrasound. Each biopsy was examined by the Pathological Anatomy Service at the Federal University of Pará following routine service. The samples were stained with hematoxylin-eosin, chromotrope aniline blue, Gomori reticulin, and Shikata's orcein.

The histopathological diagnoses followed the French METAVIR classification [17].

The assessment of the necroinflammatory histological activity index was performed by grouping the *A*_0_ and *A*_1_ patients into mild to moderate activity and the *A*_2_ and *A*_3_ patients into severe activity. The degree of fibrosis was grouped as mild to moderate (*F*_0_ to *F*_2_) and advanced (*F*_3_ and *F*_4_).

### 2.8. Data Analysis

Descriptive data analysis was performed to calculate the absolute and relative frequencies, measures of central tendency (mean, median, minimum, and maximum), and dispersion values (standard deviation).

Inferential analyses were also performed based on the characteristics of the data normality. A nonparametric ANOVA test was used for quantitative data, and Chi-square tests with or without a Yates correction, Mann-Whitney tests, Kruskal Wallis tests, and *G* tests with Williams' correction for nominal or ordinal qualitative data were used for nonparametric data. The Hardy-Weinberg equilibrium was calculated to assess the frequency of genotypes.

The odds ratio (OR) and its respective 95% confidence interval (95% CI) were calculated to measure the degree of association between the genotypes and virus infections. The programs Epi Info 3.5.3 [18] and BioEstat 5.3 [19] were used to perform the analyses, and a level of *P* ≤ 0.05 was established to reject the null hypothesis.

### 2.9. Ethical Considerations

All the patients in the study were analyzed according to the precepts of the Declaration of Helsinki and the Nuremberg Code, respecting the regulatory guidelines and rules for Research Involving Humans, according to Resolution 196/96 of the National Health Council. This project was approved by the Committee for Ethics in Research at the Holy House of Mercy Foundation of Pará.

## 3. Results 

Of the 65 individuals chronically infected with HBV, 67.7% were male and had a median age of 48 years. In total, 63.1% of these patients were from the metropolitan area, and 36.9% were from rural areas. In addition, 78.5% of the patients were HBeAg negative.

In total, 40% of the patients were clinically diagnosed as inactive carriers, 41.5% as chronic hepatitis without cirrhosis, and 18.5% as chronic hepatitis with cirrhosis.

In analyzing the allelic frequency of the polymorphisms rs12979860 and rs8099917 within the IL-28B gene, both HBV cases and controls were within the Hardy-Weinberg equilibrium (*P* > 0.05). The frequencies of the rs12979860 polymorphism were similar between the infected group (32.3% CC, 41.5% CT, and 26.2 TT) and the control population (35% CC, 47.4% CT, and 17.6% TT), and the frequencies of the rs8099917 polymorphism (7.7% GG, 35.4% GT, and 56.9% TT versus 7.2% GG, 35.1% GT, and 57.7% TT) were also similar in both groups (Table 1). Therefore, there was not any significant difference between the genotype frequencies in each group.

The association analysis between the clinical picture and the presence of polymorphisms as well as the laboratory characteristics, viral load, and serological status (Table 2) of the patients indicated that none of the associations between these variables were statistically significant. The allele and genotype frequencies of the rs12979860 and rs8099917 polymorphisms were similar when compared to the clinical presentation, except to the absence of the genotype GG (rs8099917) among patients with chronic hepatitis with cirrhosis.

The associations between the polymorphisms and liver histopathology were also not significant (Table 3). Regarding the rs12979860 polymorphism, the genotype CC was absent and TT present with frequency of 20% among patients with degree of fibrosis 3 to 4. Similarly, the data to rs8099917 polymorphism showed frequency of 18.7% to genotype TT among patients with inflammatory activity from 2 to 3. On the other hand, the genotype GG was present only in patients with inflammatory activity from 0 to 1.

A total of seven haplotypes were observed to patients and control groups (Table 4). The haplotype CCTT was the most frequent in both groups. But the haplotype association analysis of the polymorphisms between infected patients and the control group showed absence of association (Table 4).

## 4. Discussion

The infected study population in this study was predominately male (67.69%) and averaged above 40 years of age, which is similar to recent studies [3, 20].

The genotypic and allelic proportions of the rs12979860 polymorphism were not significantly different, possibly due to the sample size of the infected group and the racial composition of the population. The CT genotype (41.54%) was predominant in this group. Conversely, the predominant genotype in the literature is CC, especially in study populations with relatively little ethnic mixing [3, 21, 22].

Consistent with this study, several studies have reported no differences between the genotypic and allelic frequencies of the rs12979860 polymorphism of infected and control groups [20, 22]. However, the slight predominance of the C allele (53.1% for HBV and 58.8% in controls) found in this study was also seen in another study comparing chronically infected patients and patients with self-limiting infections in the USA [22].

Recent studies examining the rs8099917 polymorphism have not shown any significant data for genotypic and allelic frequencies. However, there was a predominance of the TT genotype upon comparing groups of chronically infected and cured patients [16], including larger groups, such as those with chronic HBV, hepatocellular carcinoma, self-limiting infections, and healthy control populations [23]. Consistent with the current literature, this study showed that there were no significant differences between frequencies (*P* = 0.9915 for the genotypes and *P* = 1.00 for the alleles) and that the TT genotype was prevalent in the population.

There were no significant associations between the polymorphisms rs12979860 and rs8099917 and the clinical presentations of the examined patients (*P* = 0.3654). The associations between clinical manifestations and these polymorphisms have been the subject of several studies, including associations with cirrhosis. However, the majority of these studies have been unable to identify any relationships [21]. Occasionally, the polymorphisms are not even addressed, especially in the case of the latter polymorphism.

Although this study did not find any significant associations, it offers important and valuable information on the associations between the liver histopathology of infected patients using the METAVIR score of inflammatory activity, the degree of hepatic fibrosis, and these polymorphisms (rs12979860 and rs8099917). There are no studies in the literature that have addressed this specific aspect when comparing the data for these different populations.

There were also no associations between any of the laboratory data and the genotypes of the two polymorphisms. Few studies have shown an association between chronic HBV patients and ALT levels. In this study, there was a higher prevalence of the CC and TT genotypes in the group with lower ALT levels for both polymorphisms [16]. However, for both the previous studies and the present study, there were no detectable differences between these genotypes and AST levels.

In this study, the viral load was also not associated with the polymorphisms rs12979860 and rs8099917 (*P* = 0.8047 and *P* = 0.4677). However, differences have been found in other studies, which showed a lower viral load in the CC genotype compared to patients with a CT + TT genotype for the first polymorphism [16] but not the second polymorphism.

Conversely, the lack of a relationship between the polymorphism haplotypes for the HBV infected cases and the cured controls was not observed for Asian populations [23]. Comparisons between the present studies and the Asian studies are difficult because the latter studies examined three polymorphisms (rs12979860 C/T, rs8099917 G/T, and rs12980275 G/A). These studies found a significant relationship between the C-T-A haplotype in patients who did not develop HCC (*P* = 0.01), but there were no significant associations between the T-G-G haplotype and progression to HCC (*P* = 0.11). Studies involving only the two polymorphisms are absent in the literature.

Studies examining the relationship between the IL-28B polymorphisms rs12679860 and rs8099917 and chronic hepatitis B remain scarce in the current literature, and research related to this subject has only been published recently. This study is a pioneer in the field by analyzing a regional sample. Therefore, it is important to determine the implications of these new findings in terms of HBV infection.

## Figures and Tables

**Table 1:** Genotypic and allelic frequencies of the polymorphisms rs12979860 and rs8099917 in the IL-28B gene in HBV patients and controls.

Polymorphism	HBV group *n* (%)	Control group *n* (%)	*P* ^a^
rs12979860			
Genotypes			
CC	21 (32.3)	34 (35.0)	0.5490
CT	27 (41.5)	46 (47.4)	
TT	17 (26.2)	17 (17.6)	
Total	**65 (100.0)**	**97 (100.0)**	
Alleles			
C	69 (53.1)	114 (58.8)	0.3694
T	61 (46.9)	80 (41.2)	
Total	**130 (100.0)**	**194 (100.0)**	
	*P* = 0.1806^b^	*P* = 0.8324^b^	

rs8099917			
Genotypes			
GG	05 (7.7)	07 (7.2)	0.9915
GT	23 (35.4)	34 (35.1)	
TT	37 (56.9)	56 (57.7)	
Total	**65 (100.0)**	**97 (100.0)**	
Alleles			
G	33 (25.4)	48 (24.7)	1.00
T	97 (74.6)	146 (75.3)	
Total	**130 (100.00)**	**194 (100.0)**	
	*P* = 0.5951^b^	*P* = 0.5626^b^	

^a^Chi-square test.

^b^Hardy-Weinberg law.

**Table 2:** Association between the clinical and laboratory variables and the IL-28B gene polymorphisms in chronically infected HBV patients.

Variables	Genotypes of polymorphism rs12979860			* P *	Genotypes of polymorphism rs8099917			*P *
	CC	CT	TT		GG	GT	TT	
Clinical presentation *n* (%)				0.3654^a^				0.4820^a^
Inactive carrier	07 (33.3)	14 (51.9)	05 (29.4)		02 (40.0)	07 (30.4)	17 (46.0)	
Chronic hepatitis without cirrhosis	09 (42.9)	08 (29.6)	10 (58.8)		03 (60.0)	11 (47.8)	13 (35.1)	
Chronic hepatitis with cirrhosis	05 (23.8)	05 (18.5)	02 (11.8)		—	05 (21.5)	07 (18.9)	
Total	** 21**	** 27**	** 17**		** 05**	** 23**	** 37**	
Laboratory $\left(\overset{-}{X}\pm \sigma \right)$								
Hemoglobin (12–18 g/dL)	13.82 ± 1.34	14.09 ± 1.94	13.12 ± 2.45	0.2729^b^	14.32 ± 1.22	14.36 ± 1.94	13.30 ± 1.93	0.0994^b^
Leukocytes (4000–10000 cells/mm³)	5475.5 ± 1607.2	6156.15 ± 1333.88	5334.12 ± 1586.56	0.1520^b^	5644.0 ± 1155.03	5607.27 ± 1395.10	5796.39 ± 1654.26	0.8964^b^
Platelets (150000–400000 cells/mm³)	170428.57 ± 66981.77	190536 ± 60965.67	193758.12 ± 65504.84	0.4584^b^	212177.60 ± 41749.27	179745.46 ± 72333.07	183916.67 ± 61634.58	0.5967^b^
AST (16–40 U/mL)	67.67 ± 69.40	47.77 ± 47.52	47.21 ± 36.05	0.3683^b^	53.86 ± 52.67	42.81 ± 28.10	61.06 ± 64.21	0.4418^b^
ALT (4–32 U/mL)	78.38 ± 117.92	57.73 ± 68.34	54.14 ± 57.61	0.6250^b^	55.46 ± 49.47	57.17 ± 71.75	68.18 ± 95.69	0.8702^b^
GGT (4–366 U/mL)	50.10 ± 42.44	64.13 ± 140.15	49.72 ± 42.09	0.8543^b^	59.06 ± 47.26	34.18 ± 15.28	67.77 ± 121.94	0.4579^b^
Viral load (U/mL)								
$\overset{-}{X}\pm \sigma$	22.549 ± 47.138	296.773 ± 1.258.700	2.556.175 ± 8.944.253	0.8047^c^	10.066.0 ± 18.663.11	357.406.77 ± 289.861.62	1.171.474.25 ± 6.104.541.01	0.4677^c^
HBeAg *n* (%)								
Positive	03 (14.3)	05 (19.2)	04 (25.0)	0.7276^a^	01 (20.0)	06 (27.3)	05 (13.9)	0.4991^a^
Negative	18 (85.7)	21 80.8)	12 (75.0)		04 (80.0)	16 (71.7)	31 (86.1)	
Total	** 21**	** 26**	** 16**		** 05**	** 22**	** 36**	

Notes: $\;\overset{-}{X}$ = arithmetic mean; *σ* = standard deviation

^a^ *G* test with Williams' correction.

^b^ANOVA

^c^Kruskal-Wallis test

AST: aspartate aminotransferase

ALT: alanine aminotransferase

GGT: gamma glutamyl transferase

HBeAg: e antigen of the hepatitis B virus

**Table 3:** Correlation between liver histopathology and the IL-28B gene genotype in chronically infected HBV patients.

Liver histopathology METAVIR (*n* = 32)	Genotypes of the polymorphism rs12979860			*P* ^a^	Genotypes of the polymorphism rs8099917			*P* ^a^
	CC *n* (%)	CT *n* (%)	TT *n* (%)		GG *n* (%)	GT *n* (%)	TT *n* (%)	
Inflammatory activity periportal/periseptal								
0 to 1	07 (77.8)	12 (92.3)	08 (80.0)	0.6058	04 (100.0)	10 (83.3)	13 (81.3)	0.5394
2 to 3	02 (22.2)	01 (7.7)	02 (20.0)		—	02 (16.7)	03 (18.7)	
Degree of fibrosis								
0 to 2	09 (100.0)	12 (92.3)	08 (80.0)	0.3137	03 (75)	11 (91.7)	15 (93.7)	0.6738
3 to 4	—	01 (7.7)	02 (20.0)		01 (25)	01 (8.3)	01 (6.3)	

Notes: conventional signal used: (—) numerical data equal to zero but not as a result of rounding.

^a^ *G* test with Williams' correction.

**Table 4:** Haplotypes of the rs12979860 and rs8099917 polymorphisms in the IL-28B gene in chronically infected HBV patients and controls.

Haplotypes	HBV group *n* (%)	Control group *n* (%)	*P* ^a^
CCGT	02 (3.0)	03 (3.1)	0.5667
CCTT	18 (27.7)	31 (31.6)	
CTGT	12 (18.5)	26 (26.5)	
CTTT	16 (24.6)	20 (21.4)	
TTGG	05 (7.7)	07 (7.2)	
TTGT	09 (13.9)	05 (5.1)	
TTTT	03 (4.6)	05 (5.1)	

Total	65 (100.0)	97 (100.0)	

Notes: conventional signal used: (—) numerical data equal to zero but not as a result of rounding.

^a^ *G* test with Williams' correction.
